# Associations Between Anemia, Cognitive Impairment, and All-Cause Mortality in Oldest-Old Adults: A Prospective Population-Based Cohort Study

**DOI:** 10.3389/fmed.2021.613426

**Published:** 2021-02-10

**Authors:** Jia Wangping, Han Ke, Wang Shengshu, Song Yang, Yang Shanshan, Cao Wenzhe, He Yao, Liu Miao

**Affiliations:** ^1^Graduate School, Chinese People's Liberation Army General Hospital, Beijing, China; ^2^Beijing Key Laboratory of Aging and Geriatrics, National Clinical Research Center for Geriatrics Diseases, Institute of Geriatrics, Second Medical Center of Chinese People's Liberation Army General Hospital, Beijing, China; ^3^Department of Military Medical Technology Support, School of Non-commissioned Officer, Army Medical University, Shijiazhuang, China; ^4^Department of Disease Prevention and Control, The First Medical Center, Chinese PLA General Hospital, Beijing, China

**Keywords:** anemia, hemoglobin, cognition, mortality, CLHLS

## Abstract

**Objective:** To evaluate the combined effects of anemia and cognitive function on the risk of all-cause mortality in oldest-old individuals.

**Design:** Prospective population-based cohort study.

**Setting and Participants:** We included 1,212 oldest-old individuals (men, 416; mean age, 93.3 years).

**Methods:** Blood tests, physical examinations, and health questionnaire surveys were conducted in 2012 were used for baseline data. Mortality was assessed in the subsequent 2014 and 2018 survey waves. Cox proportional hazards models were used to evaluate anemia, cognitive impairment, and mortality risk. We used restricted cubic splines to analyze and visualize the association between hemoglobin (Hb) levels and mortality risk.

**Results:** A total of 801 (66.1%) deaths were identified during the 6-year follow-up. We noted a significant association between anemia and mortality (hazard ratio [HR] 1.32, 95% confidence interval [CI] 1.14–1.54) after adjusting for confounding variables. We also observed a dose-response relationship between the severity of anemia and mortality (*P* < 0.001). In the restricted cubic spline models, Hb levels had a reverse J-shaped association with mortality risk (HR 0.88, 95% CI 0.84–0.93 per 10 g/L-increase in Hb levels below 130 g/L). The reverse J-shaped association persisted in individuals without cognitive impairment (HR 0.88, 95% CI 0.79–0.98 per 10 g/L-increase in Hb levels below 110 g/L). For people with cognitive impairment, Hb levels were inversely associated with mortality risk (HR 0.83, 95% CI 0.78–0.89 per 10 g/L-increase in Hb levels below 150 g/L). People with anemia and cognitive impairment had the highest risk of mortality (HR 2.60, 95% CI 2.06–3.27).

**Conclusion:** Our results indicate that anemia is associated with an increased risk of mortality in oldest-old people. Cognitive impairment modifies the association between Hb levels and mortality.

## Introduction

Anemia is a common clinical condition among older adults. The prevalence of anemia increases with age, reaching 30.7% in individuals aged 80+ years, 37% in those aged 90+ years, and >50% in centenarians (1–4). The population of oldest-old (aged ≥80 years) is rapidly growing worldwide (5). Thus, the number of the oldest-old with anemia will dramatically increase with the aging population. Anemia in older adults has significant clinical relevance. Many epidemiological studies have shown that anemia is associated with an increased risk of mortality (6–12). However, very few studies have been conducted to determine the association between anemia and mortality in oldest-old people, especially in middle-income countries.

Cognitive impairment is another condition that commonly affects older adults. This condition is burdensome and it poses a heavy burden on the public health system (13). Several studies have shown an association between cognitive impairment and increased mortality (13–15). Interestingly, anemia is a known risk factor for cognitive impairment (12, 16, 17). However, whether anemia and cognitive impairment have combined effects on mortality is still unclear. Furthermore, whether the association between anemia and mortality is influenced by cognitive status is not known. Clarifying these associations may be very relevant for clinical screening.

Thus, we conducted this study to evaluate the combined effects of anemia and cognitive impairment on the risk of all-cause mortality among oldest-old people. We also investigated whether the association between anemia and mortality is potentially modified by cognitive impairment.

## Methods

### Study Design and Participants

This study used data from the Chinese Longitudinal Healthy Longevity Survey (CLHLS), which is a prospective community-based nationwide cohort study of older people in China. Details of the CLHLS have been previously described (5, 18, 19). Briefly, this nationwide survey, conducted in 23 provinces, collected representative and widespread data to explore the determinants for healthy aging among older people in China.

Participants were recruited from the biomarker sub-study of the 6th wave (2012) of the CLHLS and followed in the 7th (2014) and the 8th (2018) wave in eight longevity areas. We included 1,427 participants aged 80 or above who had biomarker data and cognition assessments in the baseline survey. We then excluded 215 participants who were lost to follow-up. Thus, in the final analysis we included 1,212 oldest-old individuals.

The study was approved by the Ethics Committee of Duke University and Peking University (Ethics Number: IRB00001052-13074). Informed consent was signed by all participants or their legal representatives in the baseline and follow-up surveys.

### Assessment of Mortality

Mortality was ascertained during the subsequent 7th (2014) and the 8th (2018) wave of the CLHLS. The date of death was collected from local doctors or participants' close family members. The survival time was measured from the date of the first interview to the date of death or the date of the last follow-up (which occurred first).

### Anemia and Cognition Impairment

Fasting venous blood samples were drawn by medical staff, and concentration of hemoglobin (Hb) was measured with the commercially available diagnostic kits (Roche Diagnostic) in the Automatic Biochemistry Analyzer (Hitachi 7180, Tokyo, Japan). Quality control measures and more details were described previously (20). According to the World Health Organization (WHO) criteria, we defined anemia as Hb <130.0 g/L in men and <120.0 g/L in women. Anemia was further classified into three groups as follows (8): mild anemia (Hb 120–129 g/L in men and 110–119 g/L in women); moderate anemia (Hb 110–119 g/L in men and 100–109 g/L in women); severe anemia (Hb <110 g/L in men and <100 g/L in women). Based on the mean corpuscular volume (MCV), anemia was characterized as normocytic (MCV 80–100 fL) macrocytic (MCV >100 fL), or microcytic (MCV < 80 fL).

The Mini-Mental Status Examination (MMSE) is a sensitive, valid, and reliable instrument that is used extensively in clinical and research settings to measure cognitive impairment (21). Cognitive impairment was assessed by the Chinese version of the MMSE (Supplementary Table 1) (22), of which the validity and reliability have been verified (13, 23, 24). Based on the MMSE score combined with the educational time, cognition impairment was defined as an MMSE score <18 in illiterate participants, <21 in those with 1–6 education years, or <25 in those with over 6 education years (13, 25).

### Covariates

Data for covariates information were collected during home interviews and biochemistry tests. Based on the literature (13, 24, 26–28), we selected the following covariates: age at the baseline, sex, marital status (married and living with spouse/other), education years (0/1–6/>6), ethnicity (Han/others), smoking (yes/no), drinking (yes/no), body mass index(BMI is defined as the body mass divided by the square of the body height, and is universally expressed in units of kg/m^2^), Chronic conditions (hypertension, diabetes mellitus, chronic kidney disease (CKD), albumin, fasting blood glucose (FBG), high sensitivity c-reactive protein (hs-CRP), and lipid profile. Hypertension patients were defined as those with systolic blood pressure ≥140 mmHg or diastolic blood pressure ≥90 mmHg, or self-reporters (29). Diabetes mellitus patients were defined as those with FBG level ≥7.0 mmol/L or self-reporters (30). CKD patients were defined as those with estimated glomerular filtration rate (eGFR) <60 mL/min/1.73 m^2^ (26). The Chinese modified version of the Modification of Diet in Renal Disease (MDRD) equation was used to calculate eGFR: 175 × serum creatinine (mg/dL)^−1.234^ × age (years)^−0.179^ × 0.79 (if female) (31).

### Statistical Analyses

Baseline characteristics were presented as counts (%) for categorical variables and as means (standard deviations) or medians (interquartile range) for continuous variables. Student's *t*-tests or Mann–Whitney *U*-test were applied to compare the differences in the continuous variables of the two groups. Chi-square tests or Fisher's exact test were used to explore the differences in categorical variables. The association of anemia and cognitive function with all-cause mortality was analyzed using Cox proportional hazard models, including model one and two. We adjusted for age and sex in model one, and further controlled for more potential confounders such as education, marital status, BMI, albumin, hs-CRP, and chronic conditions in model two. We assessed the proportional hazards assumption using the Schoenfeld residuals technique and ascertained it had not been violated.

We use restricted cubic splines to analyze and visualize the association of Hb levels with mortality risk in all participants and participants with/without cognitive impairment. The likelihood ratio test was used to test for potential non-linearity (32, 33). If the associations of Hb levels with mortality were non-linear, we additionally calculated hazard ratios (HR) and 95% CI per 10 g/L increase in Hb based on a two-line piecewise linear model.

To observe the combined effect of anemia and cognition impairment on mortality, we created the four-level joint anemia/cognition impairment groups (group one: non-anemic and normal cognition, group two: anemic and normal cognition, group three: non-anemic and cognition impairment, group four: anemic and cognition impairment) and repeated the above analysis. The survival curve was estimated using the Kaplan–Meier and the log-rank test. The subgroup analyses of the association of four groups with mortality were conducted by age (80–89 years or ≥90 years) and sex. The interactions were tested to evaluate whether the combined effect was similar in different subgroups.

To test the robustness of the primary results, the following three sensitivity analyses were performed (24, 34): (1) excluding deaths in the first 6 months for alleviating potential confounding effects. (2) excluding deaths in the 1st year. (3) excluding participants with four kinds of self-reported diseases, such as cancer, respiratory disease, heart disease, cerebrovascular disease. (4) additionally, adjusting for the above four kinds of self-reported diseases.

These analyses were performed using Stata (version 16.0) and the R software (version 3.6.3). A *P* < 0.05 (2-tailed) was considered statistically significant.

## Results

### Baseline Characteristics

Table 1 shows the baseline characteristics of the participants. The mean age (standard deviation) of the 1,212 participants was 93.3 (8.1) years, and 34.3% of the participants were male. The prevalence of anemia was 58.7% (males, 59.0%; females, 57.9%). There were significant differences (*P* < 0.05) between the participants who had anemia and those who did not have anemia in terms of age, diabetes mellitus, CKD, BMI, eGFR, albumin, glucose, total cholesterol.

**Table 1:** General characteristics of the 1,212 participants with and without anemia.

**Characteristic**	**Overall**	**Without anemia**	**With anemia**	***P*-value**
	**(*n* = 1,212)**	**(*n* = 501)**	**(*n* = 711)**	
Age, mean (SD)	93.3 (8.1)	91.9 (8.4)	94.3 (7.6)	<0.001
Male, %	416 (34.3)	175 (34.9)	241 (33.9)	0.755
Married and living with spouse, %	245 (20.2)	112 (22.4)	133 (18.7)	0.137
**Education time, years,%**				
0	945 (78.0)	375 (74.9)	570 (80.2)	0.048
1–6	195 (16.1)	96 (19.2)	99 (13.9)	
>6	72 (5.9)	30 (6.0)	42 (5.9)	
Han ethnicity, %	1,118 (92.2)	466 (93.0)	652 (91.7)	0.464
Current smoker, %	126 (10.5)	58 (11.7)	68 (9.6)	0.294
Current drinker, %	150 (12.4)	79 (15.8)	71 (10.0)	0.003
Hypertension, %	737 (60.8)	302 (60.3)	435 (61.2)	0.797
Diabetes mellitus, %	97 (8.0)	50 (10.0)	47 (6.6)	0.043
CKD, %	233 (19.2)	46 (9.2)	187 (26.3)	<0.001
Cognitive impairment, %	421 (34.7)	175 (34.9)	246 (34.6)	0.954
Hemoglobin, g/L, mean (SD)	118.78 (20.72)	136.81 (14.52)	106.07 (13.88)	<0.001
MMSE score, mean (SD)	24.00 (12.00–28.00)	24.00 (14.00–28.00)	24.00 (12.00–28.00)	0.505
BMI, kg/m^2^, mean (SD)	20.38 (3.88)	21.23 (3.77)	19.74 (3.85)	<0.001
eGFR, mL/min/1.73 m^2^, mean (SD)	79.46 (63.79–98.36)	85.23 (72.76–102.36)	73.91 (59.10–93.59)	<0.001
Albumin, g/L, mean (SD)	39.27 (4.94)	39.92 (4.84)	38.80 (4.96)	<0.001
Hs-CRP, mg/L, median (IQR)	1.01 (0.41–2.97)	0.90 (0.42–0.62)	1.08 (0.39–3.44)	0.319
Glucose, mmol/L, mean (SD)	4.67 (2.04)	5.02 (2.31)	4.43 (1.79)	<0.001
Total cholesterol, mmol/L, mean (SD)	4.23 (0.97)	4.43 (0.92)	4.09 (0.99)	<0.001
Triglycerides, mmol/L, mean (SD)	0.93 (0.53)	0.96 (0.55)	0.90 (0.52)	0.062

*SD, standard deviations; CKD, chronic kidney disease; MMSE, Mini-Mental State Examination; BMI, body mass index; eGFR, estimated glomerular filtration rate; Hs-CRP, high-sensitivity C-reactive protein; IQR interquartile range*.

### Anemia and All-Cause Mortality

A total of 801 (66.1%) deaths were identified (men, 268; women, 533) during the 6-year follow-up. Participants who had anemia had a significantly higher mortality rate than those who did not (68.8 and 62.3%, respectively; *P* = 0.019). Table 2 presents the association between anemia and mortality. We noted a significant association between anemia and mortality (*HR* = 1.32; 95% CI, 1.14–1.54) after adjusting for confounding variables in Model two. Furthermore, participants who had severe anemia had a 51% (95% CI, 1.23–1.85) higher risk of mortality than those who did not have anemia. A dose-response relationship between the severity of anemia and mortality was also observed (*P* < 0.001). In addition, the association between anemia and mortality remained similar in normocytic, macrocytic, and microcytic anemia.

**Table 2:** Hazard ratios for the association between anemia and all-cause mortality.

**Variable**	**Crude model**		**Model 1**		**Model 2**	
	**HR (95% CI)**	***P*-value**	**HR (95% CI)**	***P*-value**	**HR (95% CI)**	***P*-value**
**Anemia**						
No (*n* = 501)	1		1		1	
Yes (*n* = 711)	1.35 (1.17, 1.56)	<0.001	1.24 (1.07, 1.43)	0.003	1.32 (1.14, 1.54)	<0.001
**Severity of anemia**						
No (*n* = 501)	1		1		1	
Mild (*n* = 266)	1.15 (0.95, 1.38)	0.152	1.07 (0.89, 1.30)	0.457	1.16 (0.96, 1.41)	0.124
Moderate (*n* = 211)	1.34 (1.10, 1.63)	0.004	1.25 (1.02, 1.52)	0.03	1.40 (1.14, 1.72)	0.001
Severe (*n* = 234)	1.65 (1.38, 1.99)	<0.001	1.45 (1.20, 1.74)	<0.001	1.51 (1.23, 1.85)	<0.001
*P* for trend	<0.001		<0.001		<0.001	
**Classification of anemia**						
No (*n* = 501)	1		1		1	
Normocytic (*n* = 516)	1.31 (1.13, 1.53)	<0.001	1.20 (1.03, 1.40)	0.02	1.31 (1.11, 1.55)	0.001
Macrocytic (*n* = 144)	1.59 (1.28, 1.98)	<0.001	1.41 (1.13, 1.76)	0.002	1.35 (1.08, 1.70)	0.010
Microcytic (*n* = 51)	1.13 (0.79, 1.63)	0.502	1.17(0.81, 1.68)	0.407	1.46 (1.01, 2.12)	0.046

*Model 1: adjusted for age, sex. Model 2: further adjusted for education, marital status, ethnicity, current smoker, current drinker, body mass index, cognitive impairment, albumin, high-sensitivity C-reactive protein, and chronic conditions (hypertension, diabetes mellitus, and chronic kidney disease)*.

### Hb Levels and All-Cause Mortality

**Figure 1:**
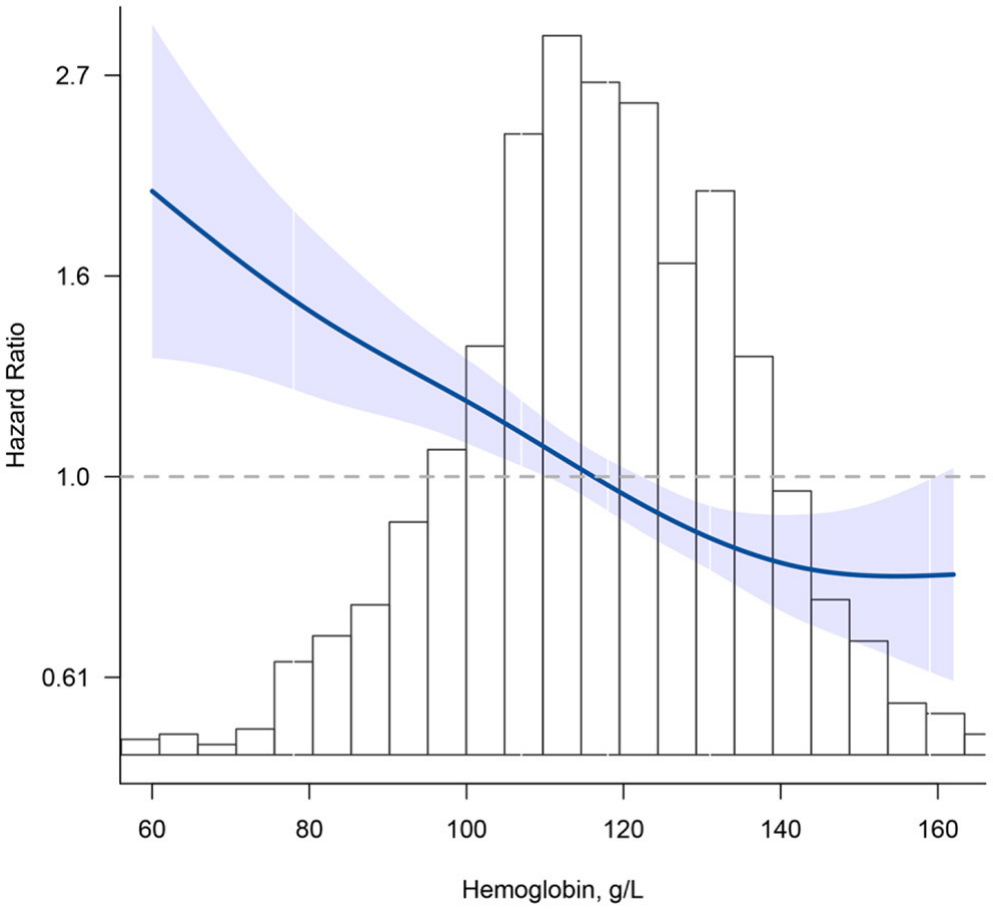
Association between hemoglobin (Hb) and all-cause mortality. Hazard ratios are indicated by blue solid lines and 95% CIs by shaded areas. Histogram represents the distribution of Hb. Model was adjusted for age, sex, education, marital status, ethnicity, current smoker, current drinker, body mass index, cognitive impairment, albumin, high-sensitivity C-reactive protein, and chronic conditions (hypertension, diabetes mellitus, and chronic kidney disease) using restrict cubic splines.

Figure 1 shows the smoothed spline curve of HRs for Hb as continuous variables. Hb levels and mortality showed a nearly reverse J-shaped relationship (*P* for non-linearity < 0.001). Below 130 g/L, the HR per 10 g/L increase in Hb levels was 0.88 (95% CI, 0.84–0.93).

**Figure 2:**
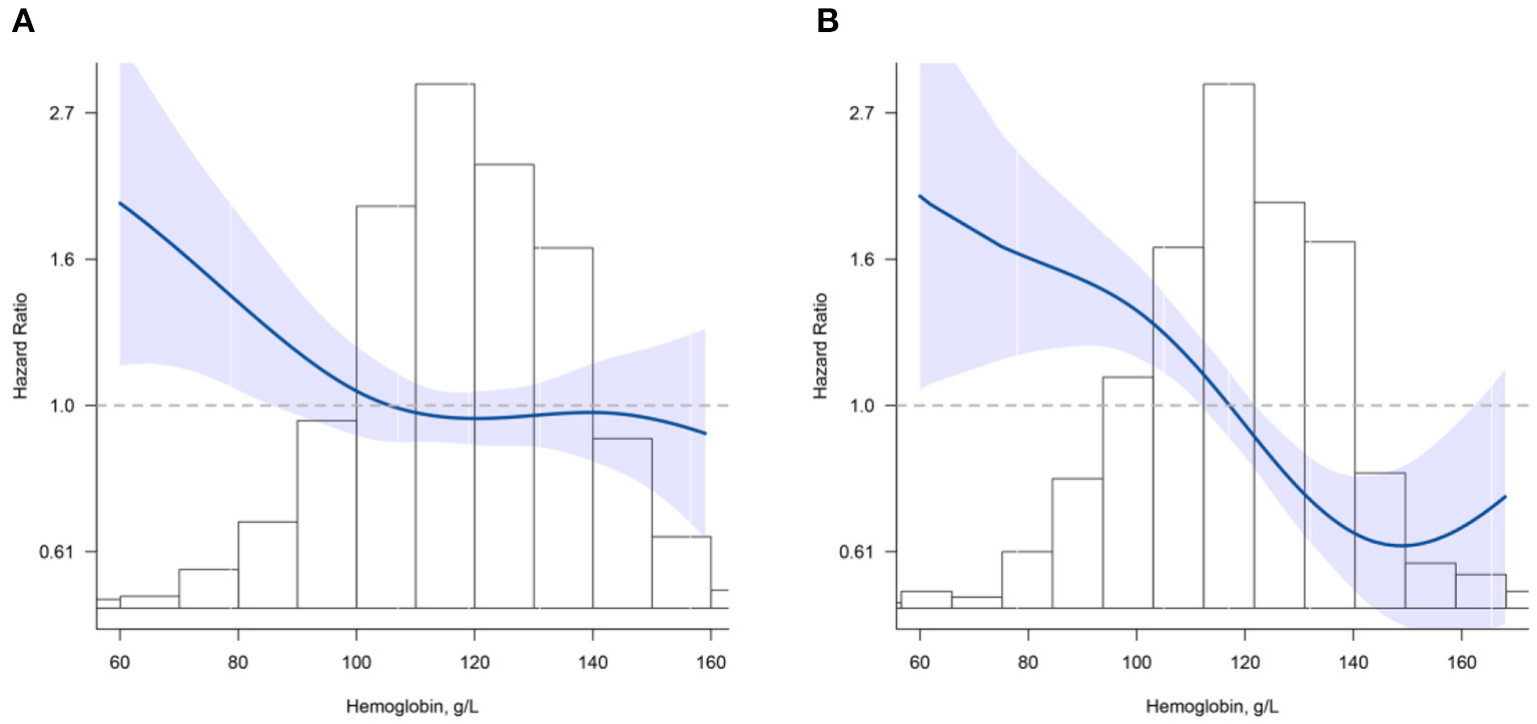
Association between hemoglobin (Hb) and all-cause mortality according to cognition impairment. **(A)** normal cognitive group; **(B)** cognition impairment group. Hazard ratios are indicated by blue solid lines and 95% CIs by shaded areas. Histogram represents the distribution of Hb. Model was adjusted for age, sex, education, marital status, ethnicity, current smoker, current drinker, body mass index, albumin, high-sensitivity C-reactive protein, and chronic conditions (hypertension, diabetes mellitus, and chronic kidney disease) using restrict cubic splines.

The shape of the curve for the association between Hb concentration and mortality was significantly modified by cognitive function (all *P* for non-linearity < 0.001) (Figure 2). For participants without cognitive impairment, there was a steep decrease in the risk of mortality with increasing Hb for concentrations <110 g/L (*HR* = 0.88; 95% CI, 0.79–0.98); for Hb concentrations above 110 g/L, the relationship was flattened (*HR* = 0.99; 95% CI, 0.92–1.06) (Figure 2A). For participants who had cognitive impairment, Hb levels were inversely associated with mortality for concentrations <150 g/L (*HR* = 0.83; 95% CI, 0.78–0.89); a positive but not significant association was noted for concentrations above 150 g/L (*HR* = 1.23; 95% CI, 0.99–1.53) (Figure 2B).

### Combined Effect of Anemia and Cognitive Status on Mortality

**Figure 3:**
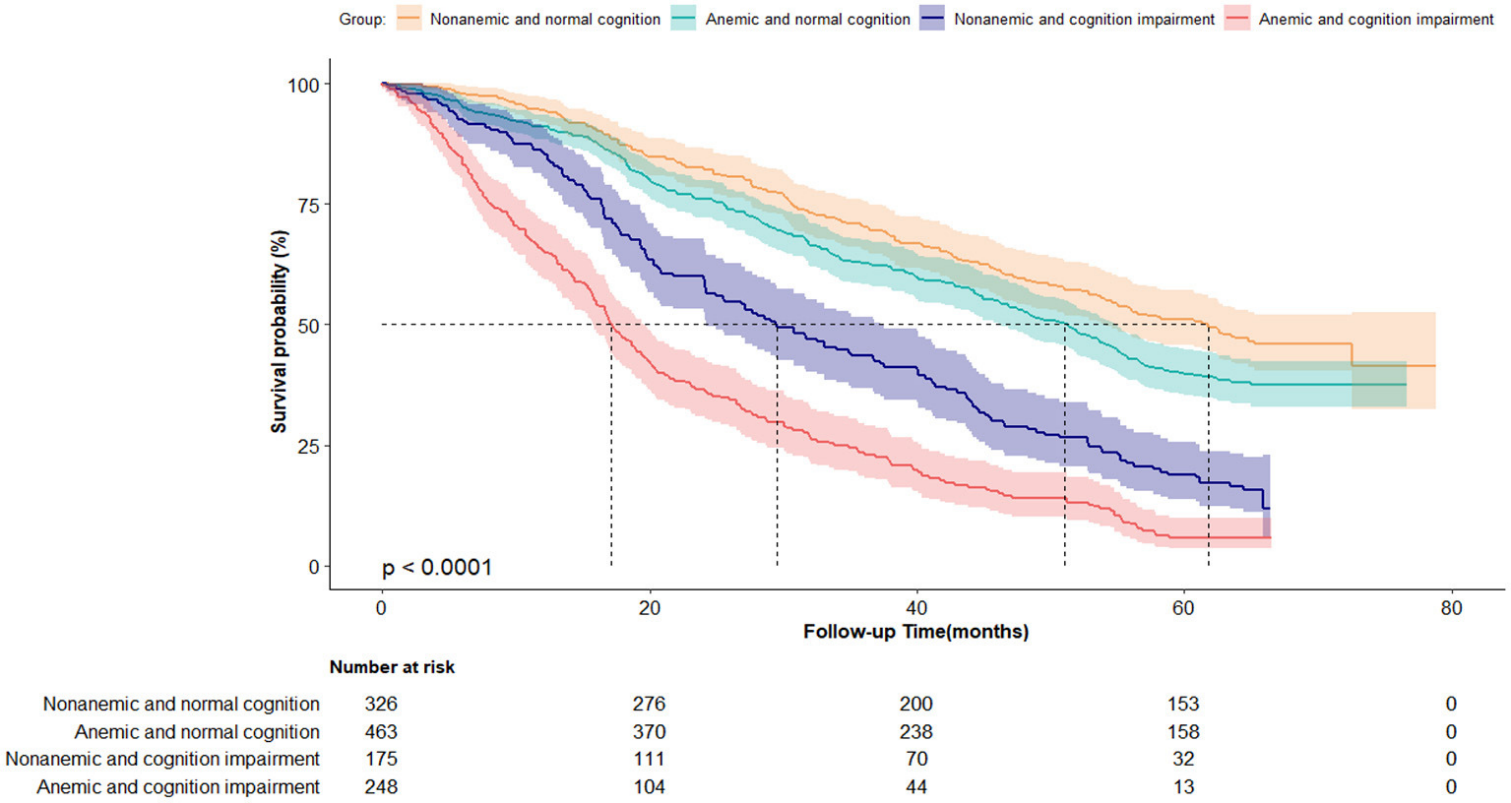
Kaplan-Meier survival curves by the 4-level joint anemia/cognition impairment groups in oldest-old adults (*N* = 1,212). Log-rank *P* < 0.0001.

Figure 3 shows the survival curves for four categories of participants stratified according to their anemia and cognitive function records at baseline. The median survival durations the four anemia/cognition impairment groups were 61.8, 51.1, 29.4, and 17.2 months, respectively. Compared with individuals who did not have anemia and cognitive impairment, people who had anemia and cognitive impairment had more than double the mortality risk (adjusted *HR* = 2.60; 95% CI, 2.06–3.27) (Table 3). In the cognitive impairment subgroup, we noted a significant association between anemia and mortality (*HR* = 1.86; 95% CI, 1.48–2.34) after adjusting for the confounding variables in Model two (Table 4). Significant interaction between anemia and cognitive impairment was observed in terms of mortality risk (*P* = 0.005).

**Table 3:** Hazard ratios for the combined associations of anemia and cognitive impairment with all-cause mortality.

**Group**	**Crude model**		**Model 1**		**Model 2**	
	**HR (95% CI)**	***P*-value**	**HR (95% CI)**	***P*-value**	**HR (95% CI)**	***P*-value**
Non-anemic and normal cognition (*n* = 326)	1		1		1	
Anemic and normal cognition (*n* = 463)	1.28 (1.05, 1.56)	0.013	1.14 (0.94, 1.39)	0.195	1.06(0.86, 1.30)	0.609
Non-anemic and cognition impairment (*n* = 175)	2.42 (1.93, 3.02)	<0.001	1.69 (1.33, 2.15)	<0.001	1.53 (1.20, 1.96)	<0.001
Anemic and cognition impairment (*n* = 248)	4.21 (3.43, 5.16)	<0.001	2.85 (2.28, 3.56)	<0.001	2.60 (2.06, 3.27)	<0.001
*P* for trend	<0.001		<0.001		<0.001	

*Model 1: adjusted for age, sex. Model 2: further adjusted for education, marital status, ethnicity, current smoker, current drinker, body mass index, cognitive impairment, albumin, high-sensitivity C-reactive protein, and chronic conditions (hypertension, diabetes mellitus, and chronic kidney disease)*.

**Table 4:** Hazard ratios for the association between anemia and all-cause mortality by cognition impairment or not.

**Group**	**Crude model**		**Model 1**		**Model 2**	
	**HR (95% CI)**	***P*-value**	**HR (95% CI)**	***P*-value**	**HR (95% CI)**	***P*-value**
**Normal cognition**						
No anemia (*n* = 326)	1		1		1	
Anemia (*n* = 463)	1.29 (1.06, 1.56)	0.01	1.09 (0.90, 1.33)	0.39	1.13 (0.85, 1.50)	0.39
**Cognitive impairment**						<0.001
No anemia (*n* = 175)	1		1		1	
Anemia (*n* = 248)	1.72 (1.39, 2.12)	<0.001	1.70 (1.48, 2.34)	<0.001	1.86 (1.48, 2.34)	<0.001

*Model 1: adjusted for age, sex. Model 2: further adjusted for education, marital status, ethnicity, current smoker, current drinker, body mass index, albumin, high-sensitivity C-reactive protein, and chronic conditions (hypertension, diabetes mellitus, and chronic kidney disease)*.

**Figure 4:**
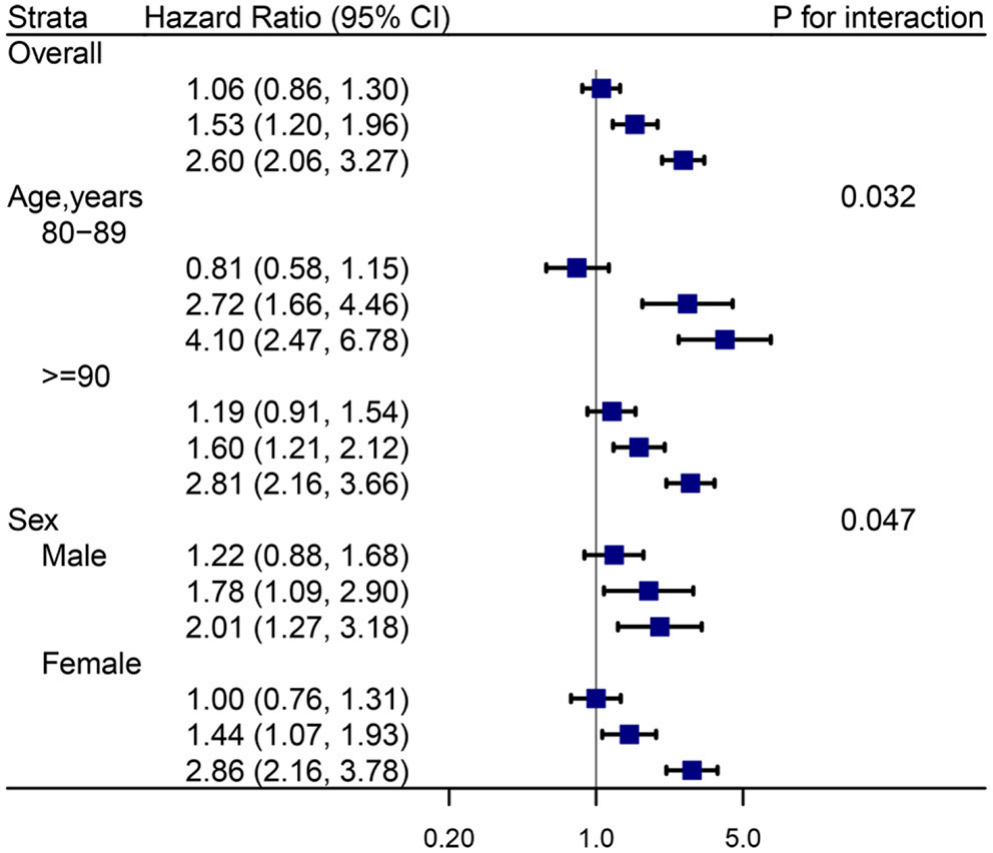
Hazard ratios (HRs) for the combined associations of anemia and cognition impairment with mortality according to age and sex subgroups. All models were adjusted for age (not in age subgroup) and sex (not in sex subgroup), education, marital status, ethnicity, current smoker, current drinker, body mass index, cognitive impairment, albumin, high-sensitivity C-reactive protein, and chronic conditions (hypertension, diabetes mellitus, and chronic kidney disease). the estimated HRs for each group are compared with individuals who did not have anemia and cognition impairment (*HR* = 1.0, not shown).

The stratified results for different age and sex subgroups are presented in Figure 4. Interestingly, the estimated risk of mortality was stronger in participants aged 80–90 years than participants aged ≥90 years (*P* for interaction = 0.032). This association between anemia, cognitive function, and mortality had a sex-specific between-group difference (*P* for interaction = 0.047). Regarding sensitivity analyses, there was almost no change in the combined associations between anemia, cognitive impairment, and all-cause mortality (Supplementary Table 2).

## Discussion

In this large prospective, community-based cohort of oldest-old adults, we evaluated the combined effects of anemia and cognitive impairment on the risk of mortality. We also investigated whether the association between anemia and mortality is potentially modified by cognitive impairment. After adjusting for potential confounders, we observed a dose-response relationship between the severity of anemia and all-cause mortality. Interestingly, we also found that the shape of curve indicating the association and Hb and mortality differs according to cognitive function. Participants who had anemia and cognitive impairment had a higher risk of mortality, and the association between anemia, cognitive function, and mortality was stronger among relatively younger oldest-old participants (aged 80–90 years).

Our results showed a significant association between anemia and increased mortality, a finding which is consistent with those of previous studies (6–12). In a large (*n* = 562) population-based study of people aged 85 years in Leiden, the Netherlands, it was estimated that anemia was associated with an increased risk of death (HR 1.41; 95% CI, 1.13–1.76), even after comprehensive adjustment for major confounders. However, none of these previous studies considered the modification effect of cognitive function on the association between anemia and mortality.

We found a non-linear relationship between Hb levels and mortality, which was consistent with previous studies (9, 35). Chaves et al. (9) observed a curvilinear slope of steady mortality decrease up to the Hb threshold of 139 g/L in 686 subjects aged ≥65 years. Zakai et al. (35) found a reversed J-shaped relationship between Hb levels and mortality in a prospective cohort study that included 11.2 years of follow-up of 5,888 participants aged ≥65 years. Further, the consideration of cognitive function in the present study expands on the findings of previous studies. Interestingly, we found that the shape of the curve depicting the association between Hb levels and mortality differed according to cognitive function. Our results provide evidence that supports screening for anemia and cognitive function in oldest-old people.

Anemia and cognitive impairment may be surrogate markers for mortality (36), although the cause pathway remains unclear. For people without cognitive impairment, the 110 g/L threshold of Hb levels may be more suitable for the oldest-old than the WHO criterion. However, even Hb levels were within the WHO criterion normal ranges, low-normal Hb levels when coexisting with cognitive impairment, predicted mortality during the 6-year follow-up. Many studies have shown that anemia and cognitive impairment are associated with increased mortality (6–15). People who have anemia and cognitive impairment may have a greater risk of mortality, as indicated by our results. Careful screening of Hb levels and cognitive impairment, as well as adequate treatment for underlying subclinical and clinical diseases, may be especially important for the oldest-old as a means of improving survival.

We also found that the association between anemia, cognitive function, and mortality appeared to be stronger among relatively younger oldest-old adults (aged 80–90 years) participants than among relatively older adults (aged 90 years or older). Robert et al. also observed the typical pattern of a decreasing relative risk (RR) with age in analyses of the effect of anemia on mortality, (ages 50–59, *RR* = 2.89; 60–69, *RR* = 2.13; 70–79, *RR* = 1.52; ≥80, *RR* = 1.34) (37). This may be partially due to survival bias (38). Survival bias, also commonly called selection bias due to loss to follow-up, can distort study results in geriatric populations (39). Those participants (aged ≥90 years) may represent the relatively healthier group of anemic individuals. Participants with anemia who died at younger ages are, in effect, missing from the cohort studies. Thus, this result warrants further studies of different age groups. This association had a sex-specific between-group difference. One potential explanation for this difference is that causes and underlying diseases of anemia may differ by sex (40). To explore possible explanations, it would be helpful to examine the causes of anemia in further studies.

To the best of our knowledge, this is the first study to evaluate the combined effect of anemia and cognitive function on mortality in a community-based nationwide cohort of oldest-old people. The strength of this study was that we included many oldest-old adults. This allowed for comprehensive adjustment for established and potential confounders and for the examination of the potential modification effect of cognitive impairment.

This study has several limitations. First, the blood parameters used in this study were based on one blood sample. Thus, the dynamic change in blood parameters, especially Hb levels, may potentially influence our results. Repeated measurements may be considered in future studies to minimize the potential bias of dynamic changes. Second, detailed information on the diagnoses of anemia and cause-specific mortality, such as iron, vitamin B12, and folate levels, were not available. Thus, we could not fully investigate the association between the multiple etiologies of anemia and type of death. However, we found that the association between normocytic, macrocytic, and microcytic anemia and mortality remained similar. Third, a total of 215 patients (15.1%) were lost to follow-up. However, this bias may be minor because there were no differences between the main characteristics of those lost to follow-up and other participants (sex: *P* = 0.978, age: *P* = 0.417, education: *P* = 0.377; smoking: *P* = 0.359, alcohol consumption: *P* = 0.406, BMI: *P* = 0.195). Further prospective studies with a more elaborate follow-up are needed to verify our results. Fourth, this, along with the Chinese nationality, may make the findings of this study less generalizable to other populations. Lastly, some unknown or unmeasured confounders might influence the association between anemia and mortality. However, we adjusted for common confounders in different models and the results were still robust.

## Conclusions And Implications

In summary, our findings indicate that anemia in oldest-old people is associated with an increased risk of mortality. We also found that cognitive impairment modified the association between Hb levels and mortality. This study provides evidence that indicates the importance of screening for anemia and cognitive function in oldest-old people. Further research should focus on the best way to translate the interaction between Hb levels and cognitive impairment into health-related screening tools for oldest-old people.

## Data Availability Statement

Publicly available datasets were analyzed in this study. This data can be found at: https://opendata.pku.edu.cn/dataverse/CHADS.

## Ethics Statement

The studies involving human participants were reviewed and approved by Ethics Committee of Duke University and Peking University. The patients/participants provided their written informed consent to participate in this study.

## Author Contributions

JW, LM, and HY contributed to the conception and design of the study. WS, YS, SY, and HK managed the data and provided help in the data analysis. JW, CW, and HK performed the statistical analysis and wrote the first draft of the manuscript. All authors contributed to the study design, critically reviewed draft versions and provided important intellectual content during revisions, and accept accountability for the overall work.

## Conflict of Interest

The authors declare that the research was conducted in the absence of any commercial or financial relationships that could be construed as a potential conflict of interest.

## References

[1] Bach V Schruckmayer G Sam I Kemmler G Stauder R. Prevalence and possible causes of anemia in the elderly: a cross-sectional analysis of a large European university hospital cohort. Clin Interv Aging. (2014) 9:1187–96. 10.2147/CIA.S61125 25092968 PMC4113572

[2] Wieczorowska-Tobis K Niemir Z Mossakowska M Klich-Raczka A Zyczkowska J Anemia in centenarians. J Am Geriatr Soc. (2002) 50:1311–3. 10.1046/j.1532-5415.2002.50328.x 12133036

[3] Haslam A Hausman DB Johnson MA Davey A Poon LW Allen RH. Prevalence and predictors of anemia in a population-based study of octogenarians and centenarians in Georgia. J Gerontology A Biol Sci Med Sci. (2012) 67:100–6. 10.1093/gerona/glr151 21896502 PMC3260487

[4] Jia W Wang S Liu M Yang S Cao W Han K. Anemia in centenarians: prevalence and association with kidney function. Hematology. (2020) 25:26–33. 10.1080/16078454.2019.1703448 31861969

[5] Zeng Y Feng Q Hesketh T Christensen K Vaupel JW. Survival, disabilities in activities of daily living, and physical and cognitive functioning among the oldest-old in China: a cohort study. Lancet. (2017) 389:1619–29. 10.1016/S0140-6736(17)30548-2 28285816 PMC5406246

[6] Mindell J Moody A Ali A Hirani V. Using longitudinal data from the Health Survey for England to resolve discrepancies in thresholds for haemoglobin in older adults. British J Haematol. (2013) 160:368–76. 10.1111/bjh.12121 23151145

[7] Izaks GJ Westendorp RG Knook DL. The definition of anemia in older persons. JAMA. (1999) 281:1714–7. 10.1001/jama.281.18.1714 10328071

[8] den Elzen WP Willems JM Westendorp RG de Craen AJ Assendelft WJ Gussekloo J. Effect of anemia and comorbidity on functional status and mortality in old age: results from the Leiden 85-plus Study. CMAJ. (2009) 181:151–7. 10.1503/cmaj.090040 19635749 PMC2717683

[9] Chaves PH Xue QL Guralnik JM Ferrucci L Volpato S Fried LP. What constitutes normal hemoglobin concentration in community-dwelling disabled older women? J Am Geriatr Soc. (2004) 52:1811–6. 10.1111/j.1532-5415.2004.52502.x 15507056

[10] Kikuchi M Inagaki T Shinagawa N. Five-year survival of older people with anemia: variation with hemoglobin concentration. J Am Geriatr Soc. (2001) 49:1226–8. 10.1046/j.1532-5415.2001.49241.x 11559383

[11] Wouters H van der Klauw MM de Witte T Stauder R Swinkels DW Wolffenbuttel BHR. Association of anemia with health-related quality of life and survival: a large population-based cohort study. Haematologica. (2019) 104:468–76. 10.3324/haematol.2018.195552 30309850 PMC6395328

[12] Denny SD Kuchibhatla MN Cohen HJ. Impact of anemia on mortality, cognition, and function in community-dwelling elderly. Am J Med. (2006) 119:327–34. 10.1016/j.amjmed.2005.08.027 16564775

[13] Duan J Lv YB Gao X Zhou JH Kraus VB Zeng Y. Association of cognitive impairment and elderly mortality: differences between two cohorts ascertained 6-years apart in China. BMC Geriatr. (2020) 20:29. 10.1186/s12877-020-1424-4 31992221 PMC6988297

[14] Perna L Wahl H-W Mons U Saum K-U Holleczek B Brenner H. Cognitive impairment, all-cause and cause-specific mortality among non-demented older adults. Age Ageing. (2014) 44:445–51. 10.1093/ageing/afu188 25468013

[15] Georgakis MK Papadopoulos FC Protogerou AD Pagonari I Sarigianni F Biniaris-Georgallis S-I Comorbidity of cognitive impairment and late-life depression increase mortality: results from a Cohort of community-dwelling elderly individuals in rural Greece. J Geriatr Psychiatr Neurol. (2016) 29:195–204. 10.1177/0891988716632913 26917554

[16] Lucca U Tettamanti M Mosconi P Apolone G Gandini F Nobili A. Association of mild anemia with cognitive, functional, mood and quality of life outcomes in the elderly: the “Health and Anemia” study. PLoS ONE. (2008) 3:e1920. 10.1371/journal.pone.0001920 18382689 PMC2271152

[17] Meng F Zhang S Yu J Chen Y Luo L He F. Low hemoglobin levels at admission are independently associated with cognitive impairment after Ischemic Stroke: a multicenter, population-based study. Translational Stroke Res. (2020) 11:890–9. 10.1007/s12975-020-00785-1 32043214

[18] Lv YB Gao X Yin ZX Chen HS Luo JS Brasher MS. Revisiting the association of blood pressure with mortality in oldest old people in China: community based, longitudinal prospective study. BMJ. (2018) 361:k2158. 10.1136/bmj.k2158 29871897 PMC5987177

[19] Zeng Y. Towards deeper research and better policy for healthy aging –using the unique data of Chinese longitudinal healthy longevity survey. China Econ J. (2012) 5:131–49. 10.1080/17538963.2013.764677 24443653 PMC3893304

[20] Shi XM Yin ZX Qian HZ Zhai Y Liu YZ Xu JW. A study on chronic diseases and other related health indicators of centenarians in longevity areas in China. Chin J Prev Med. (2010) 44:101–7. 10.3760/cma.j.issn.0253-9624.2010.02.004 20388328

[21] Pangman VC Sloan J Guse L. An examination of psychometric properties of the mini-mental state examination and the standardized mini-mental state examination: implications for clinical practice. Appl Nurs Res. (2000) 13:209–13. 10.1053/apnr.2000.9231 11078787

[22] Zhang Z. Gender differentials in cognitive impairment and decline of the oldest old in China. J Gerontol B Psychol Sci Soc Sci. (2006) 61:S107–15. 10.1093/geronb/61.2.S107 16497961

[23] An R Liu GG. Cognitive impairment and mortality among the oldest-old Chinese. Int J Geriatr Psychiatr. (2016) 31:1345–53. 10.1002/gps.4442 26891153

[24] Lv X Li W Ma Y Chen H Zeng Y Yu X. Cognitive decline and mortality among community-dwelling Chinese older people. BMC Med. (2019) 17:63. 10.1186/s12916-019-1295-8 30871536 PMC6419492

[25] Cui GH Yao YH Xu RF Tang HD Jiang GX Wang Y. Cognitive impairment using education-based cutoff points for CMMSE scores in elderly Chinese people of agricultural and rural Shanghai China. Acta Neurol Scand. (2011) 124:361–7. 10.1111/j.1600-0404.2010.01484.x 21303351

[26] Kurella M Chertow GM Fried LF Cummings SR Harris T Simonsick E. Chronic kidney disease and cognitive impairment in the elderly: the health, aging, and body composition study. J Am Soc Nephrol. (2005) 16:2127–33. 10.1681/ASN.2005010005 15888561

[27] Yin ZX Wang JL Lyu YB Luo JS Zeng Y Shi XM. Association between serum albumin and cognitive performance in elderly Chinese. Zhonghua Liu Xing Bing Xue za Zhi. (2016) 37:1323–6. 10.3760/cma.j.issn.0254-6450.2016.10.001 27765118

[28] Lyu Y Yin Z Luo J Shi X Zeng Y. Association between anemia and 3-year all-cause mortality among oldest old people in longevity areas in China. Zhonghua Liuxingbingxue Zazhi. (2015) 36:682–6. 10.3760/cma.j.issn.0254-6450.2015.07.004 26564693

[29] Go AS Bauman MA Coleman King SM Fonarow GC Lawrence W Williams KA. An effective approach to high blood pressure control: a science advisory from the American heart association, the American college of cardiology, and the centers for disease control and prevention. J Am Coll Cardiol. (2014) 63:1230–8. 10.1016/j.jacc.2013.11.007 24246165

[30] Tong YZ Tong NW Teng WP Mu YM Zhao JJ Shan ZY. Consensus on the prevention of type 2 diabetes in Chinese adults. Chin Med J. (2017) 130:600–6. 10.4103/0366-6999.200532 28229993 PMC5339935

[31] Ma YC Zuo L Chen JH Luo Q Yu XQ Li Y. Modified glomerular filtration rate estimating equation for Chinese patients with chronic kidney disease. J Am Soc Nephrol. (2006) 17:2937–44. 10.1681/ASN.2006040368 16988059

[32] Govindarajulu US Spiegelman D Thurston SW Ganguli B Eisen EA. Comparing smoothing techniques in Cox models for exposure-response relationships. Stat Med. (2007) 26:3735–52. 10.1002/sim.2848 17538974

[33] Lee DH Keum N Hu FB Orav EJ Rimm EB Willett WC. Predicted lean body mass, fat mass, and all cause and cause specific mortality in men: prospective US cohort study. BMJ. (2018) 362:k2575. 10.1136/bmj.k2575 29970408 PMC6028901

[34] Mao C Yuan JQ Lv YB Gao X Yin ZX Kraus VB. Associations between superoxide dismutase, malondialdehyde and all-cause mortality in older adults: a community-based cohort study. BMC Geriatr. (2019) 19:104. 10.1186/s12877-019-1109-z 30987591 PMC6466801

[35] Zakai NA Katz R Hirsch C Shlipak MG Chaves PH Newman AB. A prospective study of anemia status, hemoglobin concentration, and mortality in an elderly cohort: the cardiovascular health study. Arch Intern Med. (2005) 165:2214–20. 10.1001/archinte.165.19.2214 16246985

[36] Stauder R Valent P Theurl I. Anemia at older age: etiologies, clinical implications, and management. Blood. (2018) 131:505–14. 10.1182/blood-2017-07-746446 29141943

[37] Shavelle RM MacKenzie R Paculdo DR. Anemia and mortality in older persons: does the type of anemia affect survival? Int J Hematol. (2012) 95:248–56. 10.1007/s12185-012-1007-z 22351246

[38] Liu Z Han L Wang X Feng Q Gill TM. Disability prior to death among the oldest-old in China. J Gerontology A. (2018) 73:1701–7. 10.1093/gerona/gly010 29408957 PMC6230206

[39] Banack HR Kaufman JS Wactawski-Wende J Troen BR Stovitz SD. Investigating and remediating selection bias in geriatrics research: the selection bias toolkit. J Am Geriatr Soc. (2019) 67:1970–6. 10.1111/jgs.16022 31211407 PMC9930538

[40] Carmel R. Anemia and aging: an overview of clinical, diagnostic and biological issues. Blood Rev. (2001) 15:9–18. 10.1054/blre.2001.0146 11333135

