# Supplementary material for: Associations Between Anemia, Cognitive Impairment, and All-Cause Mortality in Oldest-Old Adults: A Prospective Population-Based Cohort Study
Source: Front Med (Lausanne). 2021 Feb 10;8:613426. doi: 10.3389/fmed.2021.613426 (PMC7902775; doi:10.3389/fmed.2021.613426)
Supplement: Supplementary file 1 [file Table_1.DOCX]

**Table S1 The Chinese Version of the Mini-Mental State Exam (MMSE)**

| Item | MMSE | Score (Total =30) |
| --- | --- | --- |
| Orientation | What time of day is it right now (morning, afternoon, evening)? | 1 |
|  | What is the animal year of this year? | 1 |
|  | What is the date (day and month) of the mid-autumn festival? | 1 |
|  | What is the season right now? | 1 |
|  | What is the name of this county or district? | 1 |
|  |  |  |
| Naming foods | Please name as many kinds of food as possible in 1 minute (1 point for each food and 7 points for those who name 7 or more foods) | 7 |
|  |  |  |
| Registration | Table, apple, cloth. Please repeat these three objects. | 3 |
|  |  |  |
| Attention and calculation | I will ask you to spend $3 from $20, then you must spend $3 from the number you arrived at and continue to spend $3 until you are asked to stop. | 5 |
|  |  |  |
| Copy a figure | The individual is asked to draw a figure of overlapping pentagons. | 1 |
|  |  |  |
| Recall | Name the three objects learned earlier (table, apple, and cloth). | 3 |
|  |  |  |
| Language | Naming pen and watch | 2 |
|  | Repeating the following sentence: “What you plant, what you will get.” | 1 |
|  | The individual is asked to follow the interviewer’s instruction: “Take the paper using your right hand, fold it in the middle using both hands, and place the paper on the floor.” | 3 |

| **Table S2 Sensitivity analyses for the combined associations of anemia and cognitive impairment with all-cause mortality** | | | | | | | | | | | | |
| --- | --- | --- | --- | --- | --- | --- | --- | --- | --- | --- | --- | --- |
| Group | Excluding the participants who died in the first six months | | | Excluding the participants who died in the first years | | | Excluding the participants with 4 kinds of self-reported diseases | | | Additionally, adjusting for 4 kinds of self-reported diseases | | |
|  | HR (95% CI) | *P*-value | | HR (95% CI) | *P*-value |  | HR (95% CI) | *P*-value |  | HR (95% CI) | *P*-value |  |
| Nonanemic and normal cognition | 1 |  |  | 1 |  |  | 1 |  |  | 1 |  |  |
| Anemic and normal cognition | 1.032 (0.834, 1.275) | 0.8 |  | 1.033 (0.828, 1.289) | 0.772 |  | 1.095 (0.862, 1.391) | 0.457 |  | 1.072 (0.868, 1.323) | 0.518 |  |
| Nonanemic and cognition impairment | 1.485 (1.148, 1.920) | 0 |  | 1.504 (1.148, 1.969) | 0.003 |  | 1.465 (1.097, 1.958) | 0.01 |  | 1.511 (1.174, 1.945) | 0.001 |  |
| Anemic and cognition impairment | 2.434 (1.910, 3.102) | <0.001 | | 2.143 (1.647, 2.787) | <0.001 |  | 2.691 (2.056, 3.523) | <0.001 |  | 2.545 (2.012, 3.220) | <0.001 |  |
| P for trend | <0.001 |  |  | <0.001 |  |  | <0.001 | |  | <0.001 |  |  |

HR: hazard ratio; CI: confidence interval

Sensitivity analyses were based on model2 for primary analysis.
